# Modelling, characterization, and applications of silicon on insulator loop terminated asymmetric Mach Zehnder interferometer

**DOI:** 10.1038/s41598-022-07449-0

**Published:** 2022-03-04

**Authors:** Raghi S. El Shamy, Abdelrahman E. Afifi, Mohamed M. Badr, Mohamed A. Swillam

**Affiliations:** 1 Department of Physics, The American University in Cairo, New Cairo, 11835 Egypt; 2 Electrical and Computer Engineering Department, The University of British Columbia, Vancouver, BC V6T 1Z4 Canada

**Keywords:** Electrical and electronic engineering, Integrated optics

## Abstract

This work presents a loop terminated asymmetric Mach–Zehnder interferometer (LT-aMZI) structure based on the widespread silicon-on-insulator (SOI) platform. Four different path length differences of the LT-aMZI, which correspond to free spectral ranges (FSR) from 0.8 to 6.4 nm, are designed. These designs are compared to the common asymmetric Mach–Zehnder interferometer (C-aMZI) and are shown to be more compact. These devices are suitable for optical filtering as well as wavelength demultiplexing (WDM) applications. A compact analytical model is derived that accurately describe the operation of the LT-MZI devices. The designs are then fabricated using Electron Beam Lithography (EBL) and characterized. The experimental data show good agreement when compared to the simulation results. To our knowledge, this is the first time LT-aMZI fabrication and characterization. Moreover, the LT-MZI spectrum can be tuned not only by the interferometer arms phase difference like C-MZI, but also by using its directional couplers coefficients, forming a spectral tunable filter. Finally, we determine the performance parameters of optical sensors and modulators and show that our proposed LT-MZI structure will enhance the sensor figure of merit (FOM) and modulator speed, power consumption and V_π_ × L compared to C-MZI. A comparison between symmetric and asymmetric MZI sensors and the advantage of the latter is also mentioned.

## Introduction

The rapid growth of Si-photonic industry over the past two decades, driven by the huge investments in the CMOS electronics industry, has made this new technology a potential cost effective solution for many new applications such as LIDAR systems^1^, bio-sensing^2,3^, hybrid photonic RF-ICs^4^, high speed and microwave signal processing^5,6^, etc. Silicon on Insulator (SOI) is a widespread silicon photonics technology which supports submicron optical waveguides due to its high refractive index contrast. This platform supports many other optical components and devices such as Y-junctions, couplers, interferometers, gratings and resonators^7–11^ which form the building blocks of many photonic circuits and systems^12,13^.

One of the most widely used optical devices is the Mach–Zehnder Interferometer (MZI). The simplest MZI configuration consists of two Y-junctions, one acts as a beam splitter and the other as a beam combiner and two waveguide arms. MZI is used in wide range of applications from wavelength division multiplexers^14^, optical switches^15,16^ and electro-optical modulators^17–19^ to biosensors^20,21^. One of the advantages of MZI is its high immunity to temperature fluctuations when compared to resonators^22^. However, they suffer from large footprint. This large footprint become critical when many MZIs are required on the same chip and when very large length is necessary for achieving certain performance.

In this paper, we propose loop-terminated asymmetric MZI (LT-aMZI) design based on the widespread SOI technology. The LT-MZI is simply constructed from the conventional MZI with directional coupler (DC) splitter and combiner and a loop reflector^23,24^. Our structure is the integrated version of the fiber reflection MZI (FRMZI) proposed by CA Millar et al*.*^25^. This configuration allows the propagating light to travel back and forth in the interferometer arms rather than just once; resulting in smaller footprint and boost the performance of the conventional MZI in different applications. This paper is an exhaustive extension to our previously published work^26^.

By integrating the structure presented in^25^, several important advantages are added. Mass production using widespread CMOS process will result in significant reduction in the cost of such devices. In addition, easily on-chip integration of a whole system with source, detector and electronic parts allowing for compact handheld devices. Hence, integrated high performance optical sensors, electro-optical modulators and even gyroscopes using the loop reflector^23,24^ can be implemented using our structure. Similar structures to our proposed one were published previously. In^27,28^, the same LT-MZI structure was presented but in the symmetric configuration and was used as a mirror with controllable reflectivity which then used by^27^ to construct a Fabry–Perot cavity. While in^29^ Michelson interferometer was proposed employing two loop mirrors compared to just one used in LT-MZI, i.e. more compact. Also, in^30^ authors use asymmetric LT-MZI together with ring resonator to construct a Vernier sensor. Their work is based on numerical design and optimization using the transfer matrix method. Here we have derived a simple and compact analytical model that accurately describes the LT-aMZI structure rather than the complex transfer matrix method used in^30^. Using our structure, we have designed optical filters and compared these designs with the common MZI showing our designs to be more compact. Simulation of the optical filter designs have been performed using Lumerical Interconnect^31^ which is fast circuit simulation software. These LT-aMZI designs have also been fabricated and characterized for the first time, with measurements showing good matching with Interconnect simulations as well as the analytical model. In addition, unlike conventional MZIs and Michelson interferometer in^29^ the transmission spectrum of our proposed LT-MZI can be engineered using the DCs coupling coefficients and not just by the interferometer arms phase difference, which can be used as spectral tunable filter. Finally, using our compact model we show that our proposed LT-MZI structure enhance significantly the performance of optical sensors and electro-optical modulators that were using the widespread conventional MZI. Analysis also shows that the asymmetric MZI (aMZI) configuration offers an advantage over the symmetric MZI (sMZI) in sensing as both sensitivity and FOM can be engineered independently in aMZI, which is not applicable in the case of sMZI and also ring resonators.

## Results

### Structure and modeling

**Figure 1:**
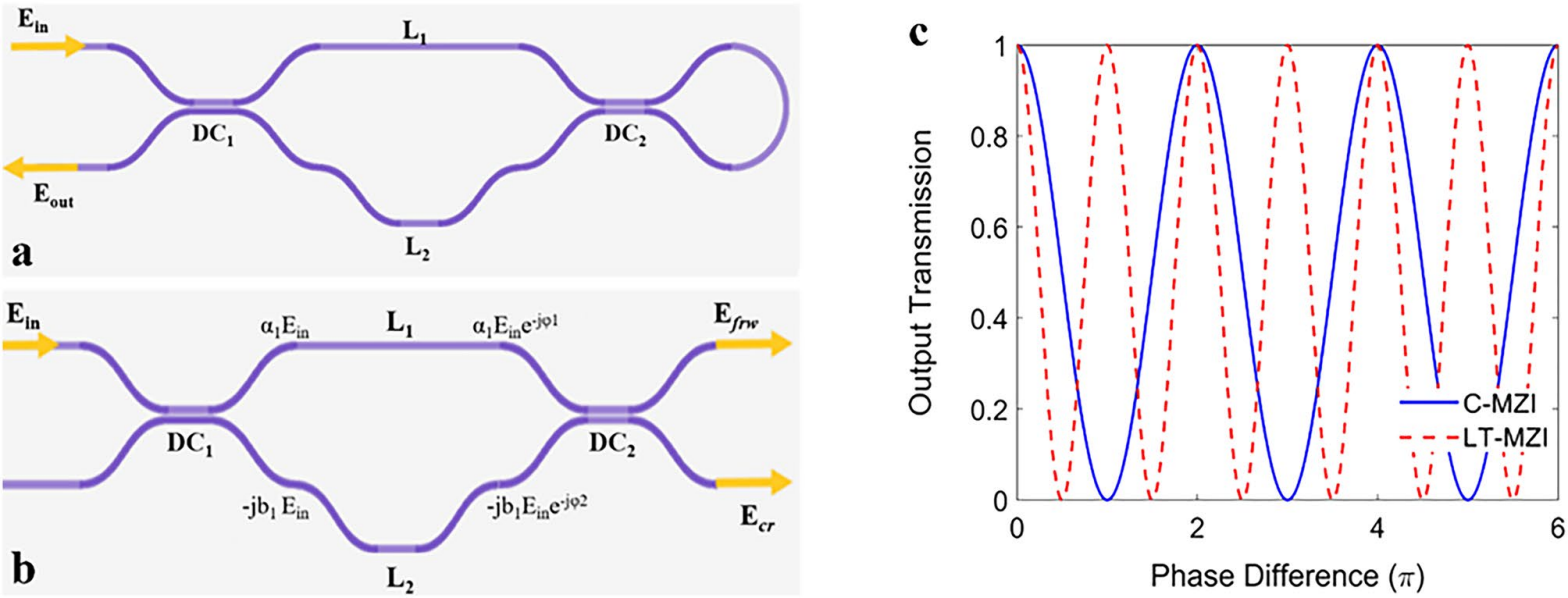
(**a**) Schematic of our proposed LT-aMZI, (**b**) schematic of C-aMZI_DC_ **c** output transmission versus interferometer arms phase difference of both LT-MZI and C-MZI with 3-dB directional couplers.

Our proposed loop terminated asymmetric Mach–Zehnder Interferometer (LT-aMZI) is shown in Fig. 1a, which is constructed from input directional coupler (DC) that split the input wave to the interferometer arms. The two interferometer arms experience different phase shift φ_1_ and φ_2_ and then another DC is used to combine the two arms waves. The outputs of the second DC are connected through a bend waveguide utilizing loop reflector as the one in^23,24^. This loop reflect the waves back to the interferometer and the output is taken from the first DC as shown in Fig. 1a. Thus, our structure is constructed of a conventional MZI (C-MZI) using DCs, denoted as C-MZI_DC_, and loop reflector. Hence, the transmission of our proposed LT-MZI can be derived using the C-MZI_DC_ forward t_frw_ and cross t_cr_ transmission coefficients shown in Fig. 1b.

The intensity transmission of our LT-MZI can be expressed as:

1 $$ T_{LT {\text{-}} MZI} = \left| {t_{LT {\text{-}} MZI} } \right|^{2} = A\left[ {\frac{H}{4} + \cos \left( {\Delta \phi } \right)} \right]^{2} $$

2a $$ \text{where} \quad A = 16a_{1}^{2} a_{2}^{2} b_{1}^{2} b_{2}^{2} $$

2b $$ \text{and} \quad H = - \frac{{a_{1} a_{2} }}{{b_{1} b_{2} }} - \frac{{b_{1} b_{2} }}{{a_{1} a_{2} }} + \frac{{a_{1} b_{2} }}{{a_{2} b_{1} }} + \frac{{a_{2} b_{1} }}{{a_{1} b_{2} }} $$

where ɑ_1_, b_1_ are the forward and cross coupling coefficients of the first DC and ɑ_2_, b_2_ are the forward and cross coupling coefficients of the second DC. Also, φ_1_ and φ_2_ are the phase shifts of the upper and lower arms of the interferometer, respectively. We here focus on asymmetric (or unbalanced) MZI, i.e. arms with different lengths L_1_ and L_2_ and with identical waveguides at both arms, i.e. β_1_ = β_2_ = β. Hence, φ_1_ = βL_1_, φ_2_ = βL_2_ and Δφ = φ_2_ − φ_1_ = βΔL with ΔL = L_2_ − L_1_ is the interferometer path length difference.

From Eq. (1) we can simply derive the main parameters of the LT-aMZI which are the resonance wavelength (λ_res_), free spectral range (FSR) and full width half maximum (FWHM) as:

3a $$ \lambda_{res} = \frac{{2n_{eff} \Delta L}}{q} $$

3b $$ FSR_{LT {\text{-}} aMZI} = \frac{{\lambda^{2} }}{{2n_{g} \Delta L}} $$

For $$a_{1} = a_{2} = b_{1} = b_{2} = \frac{1}{\sqrt 2 }$$, H = 0:

3c $$ FWHM_{LT {\text{-}} aMZI} \approx \frac{{FSR_{LT {\text{-}} aMZI} }}{\pi } $$

with q an integer number, n_eff_ and n_g_ are the waveguide mode effective index and group index, respectively. Note that, FWHM is the spectral width Δλ with transmission greater than or equal to T_max_/2, which can be derived from Eq. (1) using $T(\lambda_{res} + \frac{\Delta \lambda }{2}) = \frac{{T_{\max } }}{2}$.

Figure 1c shows the change in the output transmission due to change in the phase difference of both C-MZI and LT-MZI with ɑ_1_ = b_1_ = ɑ_2_ = b_2_ = 1/$\surd 2$, i.e. 3-dB directional couplers. It can be seen that, LT-MZI have higher rate of change compared to C-MZI, specifically two times higher. Due to this characteristic LT-aMZI can achieve the same FSR of the C-aMZI using half its path length difference (ΔL). Also, the FWHM of the LT-aMZI is half that of C-aMZI with the same path length difference, because the FSR of the LT-aMZI is half that of C-aMZI. Reducing the FWHM of the interferometer is useful in many applications such as sensors and modulators.

### Designs and simulations

**Figure 2:**
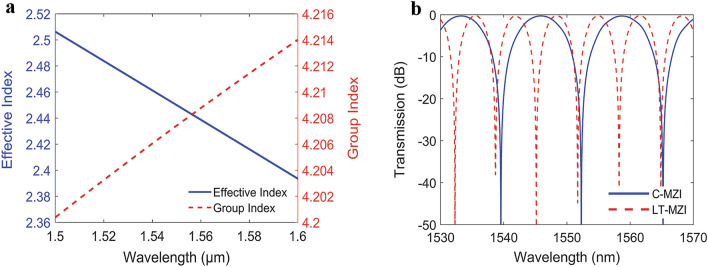
(**a**) Effective index and group index versus wavelength for the fundamental TE like mode of the (500nmx220nm) strip waveguide obtained from mode solver and (**b**) output transmission spectra versus wavelength of both LT-aMZI and C-aMZIY for path length differences ΔL = 44.7 µm.

We use the quasi-TE mode of the standard SOI strip waveguide with width w = 500 nm and thickness h = 220 nm and silicon dioxide cladding to construct our interferometer designs. We have performed modal analysis for this waveguide’s TE mode using finite difference eigenmode solver^32^ to get its effective and group index over the wavelength, see Fig. 2a. This waveguide width, w = 500 nm, is used as it exhibits the least bend and roughness loss while still supporting a single mode.

Using our proposed LT-aMZI, we demonstrate four different designs with different path length differences (ΔL) shown in Table 1. These devices are suitable for optical filtering as well as wavelength de-multiplexing (WDM) applications. We compare these designs with C-aMZI of the same path length difference ΔL. When simulated the C-aMZI y-junctions rather than DCs were used to split and combine the power to and from the interferometer arms, denoted C-aMZI_Y_. This C-aMZI_Y_ is used when comparing with our LT-aMZI as it is more practical due to the even splitting of the y-junction over broadband. Lumerical Interconnect software tool was mainly used in devices simulations as it is more computationally efficient when compared to 3D FDTD simulations. Interconnect uses scattering matrices of optical components to determine the transfer function of photonic integrated circuits (PICs). In addition, some results have been verified with analytical modeling Eq. (1) showing a very good matching.

**Table 1:** LT-aMZI designs dimensions and FSR.

	w_1_ = w_2_	ΔL	FSR
Design 1	500 nm	44.7 µm	6.4 nm
Design 2	500 nm	89.4 µm	3.2 nm
Design 3	500 nm	178.8 µm	1.6 nm
Design 4	500 nm	357.6 µm	0.8 nm

Figure 2b shows the Interconnect simulation results of our proposed LT-aMZI and the corresponding C-aMZI_Y_ simulation with ΔL = 44.7 µm. From this result we can see that, as mentioned before, for the same interferometer path length difference ΔL the FSR of the LT-aMZI is half that of the C-aMZI. The DC and y-junction used in the simulations are optimized designs from Lumerical E-Beam Compact Model Library (CML)^33^ that result in even power splitting at both outputs over 100 nm bandwidth around 1.55 µm wavelength.

### Measurements and characterization

**Figure 3:**
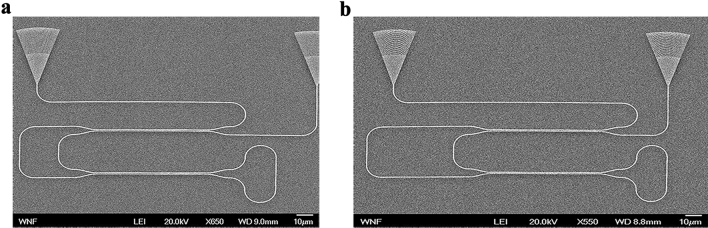
SEM photos of the fabricated LT-aMZIs: (**a**) with ΔL = 44.7 µm, (**b**) with ΔL = 89.4 µm.

The dimensions of the fabricated LT-aMZIs designs are included in Table 1. The designs were fabricated at the UW NNCI Washington Nanofabrication Facility using Electron Beam lithography^34^ on standard SOI wafers with silicon device layer of 220 nm. We fabricated three copies for each design to account for the fabrication tolerance. Figure 3a,b show SEM photos of the fabricated LT-aMZIs. Note that, the SEM photos are taken before depositing the SiO_2_ clad. As can be seen, bend waveguide loop is crucial in determining the device footprint thus, LT-MZI structure is favorable over the Michelson interferometer where two loop mirrors are needed and hence will increase the footprint significantly. A tunable laser is used to span the wavelength region from 1500 to 1600 nm. A polarization maintaining fiber array containing 4 fibers separated by 127 µm is used to couple light in and out of the chip through the grating couplers while a detector is used to measure the output power from the fibers. The GC used is also from the library in^33^. A typical SOI MZI have around 0.5 dB losses (with arms’ length in micrometer dimensions)^35,36^ and we can expect roughly that our device losses will be twice that value.

**Figure 4:**
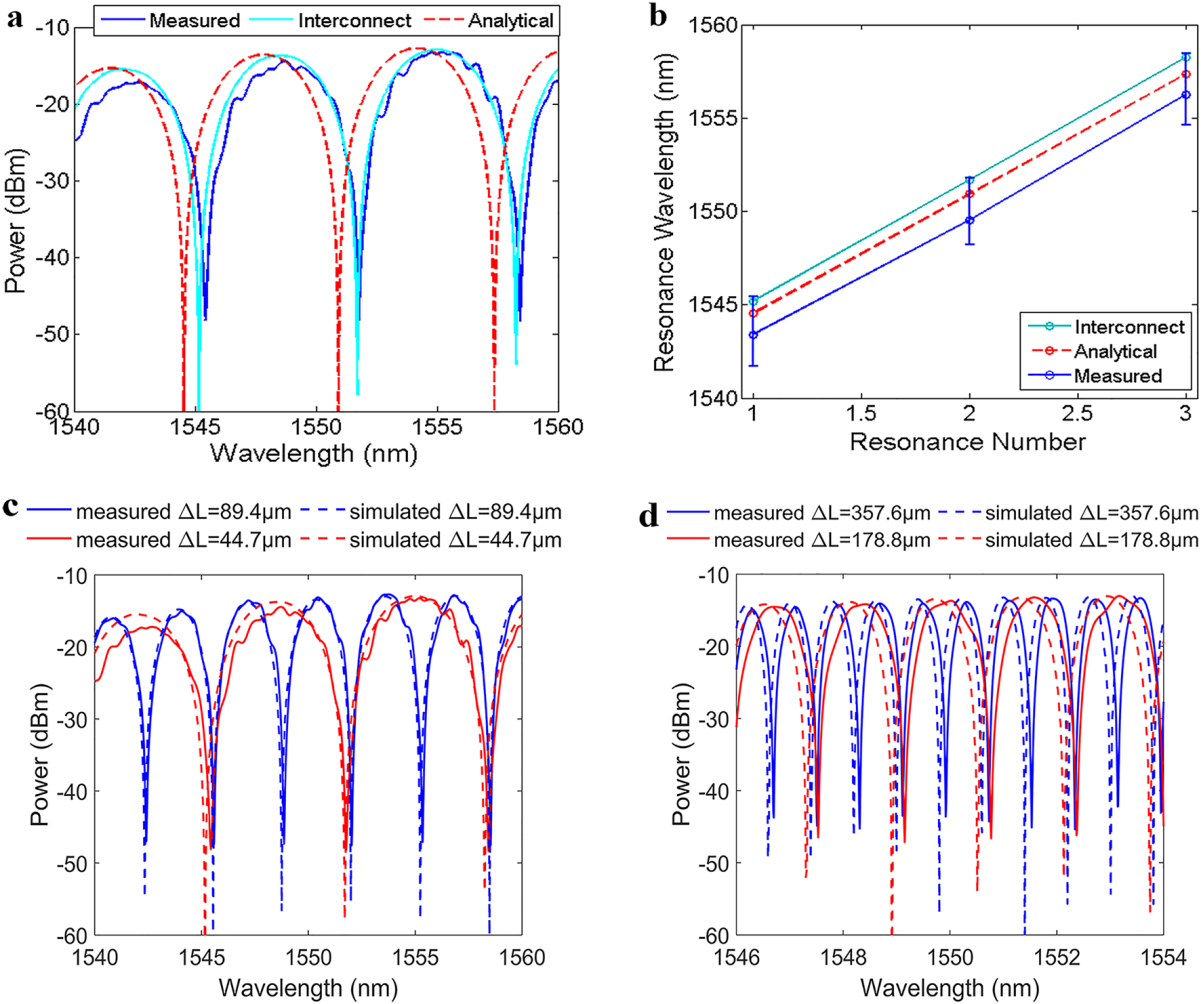
Comparison of the: (**a**) output power versus wavelength and, (**b**) resonance wavelengths from measured, Interconnect and analytical modelling of the LT-aMZI with ΔL = 44.7 µm. The measured (solid) and simulated (dotted) output power spectra versus wavelength of the LT-aMZI designs with path length differences: (**c**) ΔL = 44.7 µm (red) and ΔL = 89.4 µm (blue) and (**d**) ΔL = 178.8 µm (red) and ΔL = 357.6 µm (blue).

The measurement results of the fabricated designs are compared with different modelling methods to ensure the feasibility of the proposed LT-aMZI structure. Figure 4a shows the device output power spectrum of the fabricated and characterized LT-aMZI Design1 with ΔL = 44.7 µm together with the Interconnect simulation, and analytical model from Eq. (1). Note that we have added the device IL to the Interconnect and analytical model results. The results show very good agreement among the different modelling methods. The FSR of the different modelling methods is listed in Table 2, showing less than 2.2% difference among the different modelling methods.

**Table 2:** Calculated FSR from measurements, Interconnects, and analytical model of the LT-aMZI with ΔL = 44.7 µm.

	FSR (nm)	Difference (%)
Measured	6.490	–
Interconnect	6.440	0.770
Analytical	6.348	2.192

As mentioned above, the four designs, ΔL = 44.7 µm, 89.4 µm, 178.8 µm and 357.6 µm, were fabricated with three copies for each design to account for the fabrication tolerances. Figure 4b shows the resonance wavelengths of the LT-aMZI as calculated from the different modelling methods for the same design ΔL = 44.7 µm with error-bars showing the variations due to fabrication tolerances. Overall, it can be seen that the different modelling methods have good matching with the experimental results with deviation that is lying within the fabrication tolerances. Exact matching is not satisfied for two main reasons. Firstly, the used refractive indices for the silicon and silicon dioxide in Interconnect simulations are not exactly the same as the real refractive indices used in the fabrication of the devices. Secondly, is the dimension mismatch between the devices dimensions on the layout and the fabricated dimensions. Hence, the effective, group index of the waveguide mode, the lengths of the interferometer components and accordingly the resonant wavelengths and FSR will not be matched. We will stick to Interconnect simulations in the rest of the paper because, as mentioned above, it is computationally efficient, especially as the interferometer length is increased.

Figure 4c,d show the measured output power of the fabricated LT-aMZI designs together with the simulation results of Interconnect after adding to this simulation results the insertion loss from the measured data. The measured data are from the samples that best fit the Interconnect simulations. Table 3 shows the FSR determined from simulations and measurements showing less than 2.6% difference among them at 1.55 µm wavelength for the four designs.

**Table 3:** Calculated FSR from simulations and measurements near 1.55 µm of the four different LT-aMZI designs and the difference percentage.

	FSR simulation	FSR measurement	Difference (%)
Design 1	6.56 nm	6.66 nm	1.5
Design 2	3.24 nm	3.17 nm	2.2
Design 3	1.62 nm	1.6 nm	1.25
Design 4	0.81 nm	0.79 nm	2.53

## Discussion

### Spectral tunable filter

Another unique characteristic of our proposed LT-MZI is that its transmission spectrum can be tuned using the DCs coupling coefficients, working as spectral tunable filter. While this effect is mentioned in^30^ however, they just investigated three sets <ɑ_1_ − ɑ_2_> of DCs coefficients and they studied the effect of fabrication tolerances for this specific sets. Here, we discuss the different possible trends of the LT-MZI transmission spectrum which occur due to specific relation between DC_1_ and DC_2_ coupling coefficients and which are of interest for spectral filter applications.

**Figure 5:**
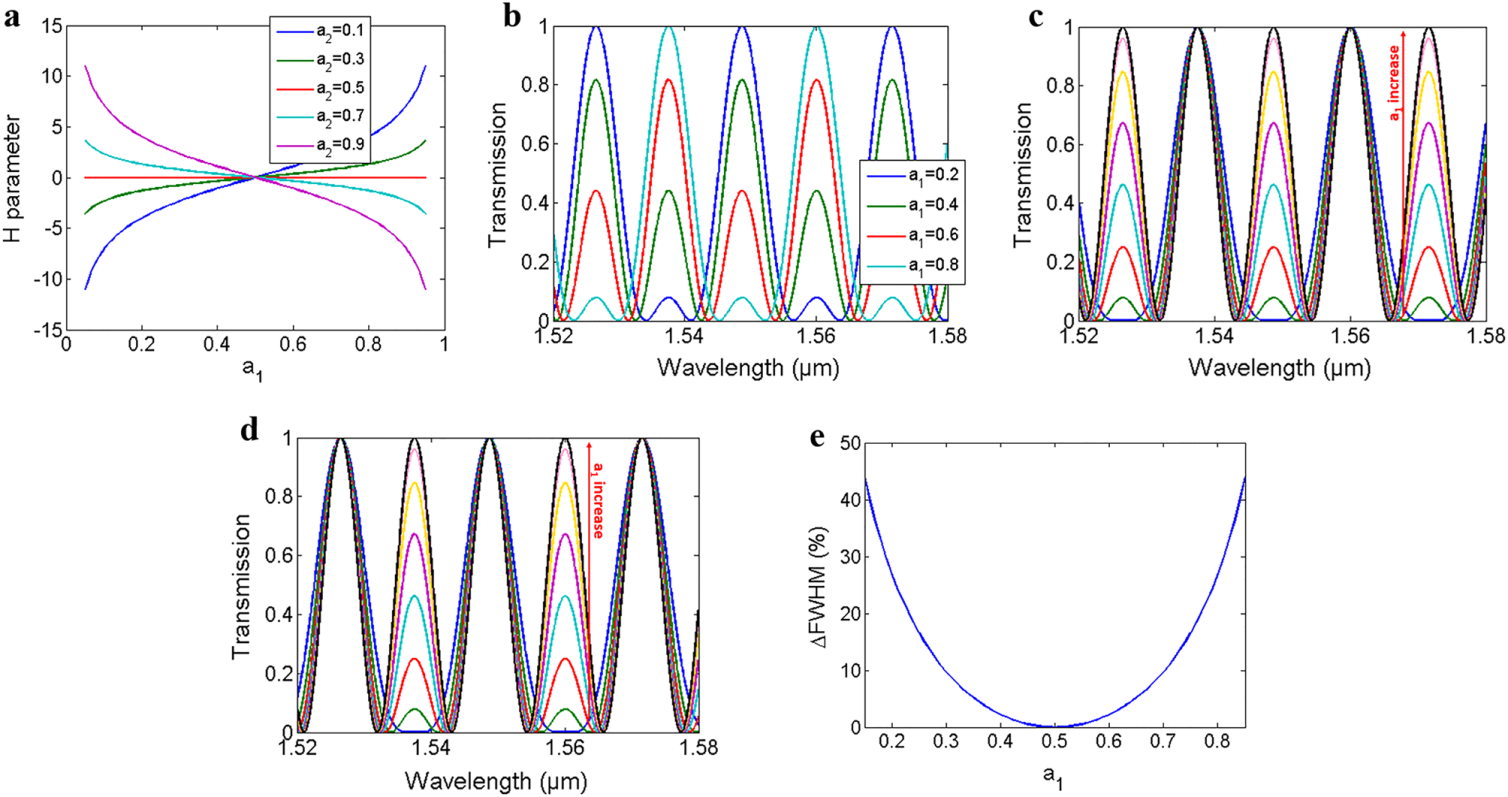
(**a**) H parameter versus a_1_ coefficient at different a_2_. Transmission spectrum versus wavelength of SOI waveguide TE mode at w = 500 nm and ΔL = 25.4 µm with: (**b**) a_2_ = 0.8 at different a_1_, (**c**) at different a_1_ with a_2_ = a_1_ and (**d**) at different a_1_ with a_2_ = 1 − a_1_ where a_1_ increase from 0.15 to 0.5 with 0.05 step as indicated by the arrow. (**e**) Percentage change of the main loop FWHM versus a_1_ for part c and d.

In the previous sections we focused on LT-MZI with 3-dB DCs (ɑ_1_^2^ = ɑ_2_^2^ = 0.5). In this case, H = 0 and the transmission spectrum reduce to cos^2^ function, see Eqs. (1) and (2). In addition, the FSR and FWHM is half that obtained using a C-MZI with the same path length difference, see Fig. 1c. However, changing the DCs coefficient from this condition will change the H parameter to non-zero values and accordingly the transmission spectrum changes. Figure 5a shows H values at different a_1_ = ɑ_1_^2^ and a_2_ = ɑ_2_^2^ with b_1_^2^ = 1 − ɑ_1_^2^ and b_2_^2^ = 1 − ɑ_2_^2^, i.e. lossless DCs. H is positive when a_1_ > 0.5 and a_2_ < 0.5 or vice versa and negative if both a_1_,a_2_ > 0.5 or a_1_,a_2_ < 0.5.

There are mainly three special cases of interest resulting in different trends of the LT-MZI output spectrum. First, changing a_1_ while a_2_ is fixed at 0.8, see Fig. 5b. It can be seen that, by sweeping DC_1_ coupling coefficients the transmission intensity of even and odd resonances are changed inversely and we can flip from a spectrum having only the even resonances to a spectrum having only the odd resonances, thus controlling the relative intensity of the even and odd resonances. Second, changing the DCs coefficients with a_1_ = a_2_, see Fig. 5c, in this case even resonances are always maximum with T = 1 while odd resonances intensity can be controlled from 0 to 1 by changing a_1_ from 0.15 to 0.5. Finally, changing DCs coefficients such that a_1_ + a_2_ = 1, see Fig. 5d, in this case odd resonances have T = 1 while even resonances intensity can be controlled. Note that, in this cases the FSR can be doubled from the case when a_1_ = a_2_ = 0.5. All the transmission spectra of Fig. 5 are obtained using SOI waveguide with w = 500 nm and ΔL = 25.4 µm.

As can be seen from the figures, in the two later cases the main loop FWHM changes while changing the DCs coefficients. FWHM is minimum when a_1_ = a_2_ = 0.5 and as a_1_ moves far from 0.5 the FWHM increase. Figure 5e shows the FWHM percentage change from the minimum value at a_1_ = a_2_ = 0.5 as a function of a_1_. We can see that for a_1_ = 0.15 the FWHM increase by 44%. Also note that, while figures shows only a_1_ varying from 0.15–0.45 the response is symmetric for a_1_ values from 0.5–0.85, i.e. a_1_ = 0.45 and a_1_ = 0.55 give the same response as they result in the same H value, see Fig. 5a.

Unlike the transfer matrix method used in^30^, this behavior can be easily interpreted using our derived closed form expression of Eq. (1). Simply, as DCs coefficients change as H parameter which is a constant shift to the cosine function changes. Accordingly, the part of the cosine below (or above) the zero changes and hence when squaring it the relative intensities of the even and odd resonances changes. Such phenomena does not exist in C-MZI or the Michelson interferometer proposed in^29^, changing the DCs coefficients in these structures will only change the transmission intensity of the whole spectrum evenly.

### Electro-optical modulator

Our proposed LT-MZI design is very promising to enhance the performance of electro-optical modulators that use C-MZI, similar to the Michelson interferometer modulator proposed in^29^. One of the main electro-optical modulator parameters is the term V_π_ × L which defines the voltage V_π_ needed to change the output power of the interferometer with length L from High to Low. Other important parameters are the energy consumption of the modulator and modulation speed (bandwidth). Modulation speed is mainly limited by the RF losses, walk-off between the electrical and optical signal and RC time constant. On the other hand, energy consumption are determined by the RF losses, capacitive loading and the 50 Ω termination resistor. For state-of-the-art modulators such as the ones based on silicon organic hybrid (SOH) platform^37–40^, the modulator’s length can be as small as 500 µm. At such compact length RF losses are reduced^37,38^ and the modulator behave as a lumped element that can be derived without the 50-Ω termination^38–40^. Accordingly, walk-off is negligible and RC time constant is the main bandwidth limitation, *f*_3dB_ = 1/2πRC^37,38^. Also, the energy consumption is reduced and determined mainly by the capacitive load^38–40^, which is proportional to CV^2^. Hence, decreasing the capacitance of such modulators will further enhance their performance.

Now assume a symmetric (balanced) interferometer, L_1_ = L_2_ = L, where the applied voltage V changes the interferometer arms effective index and hence switching the output power from high to low. For LT-aMZI and C-aMZI with $a_{1} = a_{2} = b_{1} = b_{2} = 1/\sqrt 2$ we have:

4a $$ T_{LT {\text{-}} aMZI} = \cos^{2} \left( {\Delta \phi } \right) $$

4b $$ T_{C {\text{-}} aMZI} = \cos^{2} \left( {\frac{\Delta \phi }{2}} \right) $$

Hence to switch from ON to OFF we need:

5a $$ \Delta \phi = \pi \Rightarrow \frac{{2\pi \Delta n_{eff} L_{C - MZI} }}{\lambda } = \pi \Rightarrow L_{C {\text{-}} MZI} = \frac{\lambda }{{2\Delta n_{eff} }} $$

5b $$ \Delta \phi = \frac{\pi }{2} \Rightarrow \frac{{2\pi \Delta n_{eff} L_{LT - MZI} }}{\lambda } = \frac{\pi }{2} \Rightarrow L_{LT {\text{-}} MZI} = \frac{\lambda }{{4\Delta n_{eff} }} $$

Δn_eff_ is proportional to the applied ΔV. For the same waveguide configuration and same applied voltage V, both C-MZI and LT-MZI will have the same Δn_eff_, Hence, Eq. (5) shows that the V_π_ × L term will be two times smaller for our proposed LT-MZI. This shows that LT-MZI design is more compact (L_LT-MZI_ = L_C-MZI_**/**2). On the other hand, the capacitance of the modulator expressed by Eq. (6) will also decrease to half the value of the C-MZI (C_LT-MZI_ = C_C-MZI_**/**2). Accordingly, for compact state-of-the-art modulators this will effectively reduce the energy consumption and increase the modulation speed for the LT-MZI design.

6 $$ C = \varepsilon_{0} \varepsilon_{r} \frac{h \times L}{d} $$

where ε_r_ is the permittivity of the capacitor dielectric material, d is the conducting plate separation, h is the waveguide thickness and L is the interferometer arm’s length.

### Optical sensor

The main performance parameter of optical sensors is the figure of merit (FOM) which is defined as^41^:

7 $$ FOM = \frac{{d\lambda /dn_{med} }}{FWHM} = \frac{S}{FWHM} $$

where S is the wavelength sensitivity and n_med_ is the refractive index of the sensed medium. The resonance wavelengths for LT-aMZI and C-aMZI are:

8a $$ \Delta \phi = q\pi \Rightarrow \lambda_{res,LT {\text{-}} aMZI} = \frac{2}{q}(n_{eff,sens} L_{sens} - n_{eff,ref} L_{ref} ) $$

8b $$ \frac{\Delta \phi }{2} = q\pi \Rightarrow \lambda_{res,C {\text{-}} aMZI} = \frac{1}{q}(n_{eff,sens} L_{sens} - n_{eff,ref} L_{ref} ) $$

where n_eff,sens_, L_sens_ and n_eff,ref_, L_ref_ are the effective index, arm length of the sensing and reference arm, respectively. Hence, the wavelength sensitivity of both interferometers can be derived to be:

9a $$ S_{LT {\text{-}} aMZI} = \frac{{\lambda_{res} }}{{(n_{eff,sens} L_{sens} - n_{eff,ref} L_{ref} )}}L_{sens} S_{wg} $$

9b $$ S_{C {\text{-}} aMZI} = \frac{{\lambda_{res} }}{{(n_{eff,sens} L_{sens} - n_{eff,ref} L_{ref} )}}L_{sens} S_{wg} $$

where S_wg_ = dn_eff_/dn_med_ is the sensing arm waveguide sensitivity. So, both LT-aMZI and C-aMZI configuration have the same wavelength sensitivity S for the same waveguide structure and same dimensions. This sensitivity increase as L_sens_ → (n_ref_/n_sens_) × L_ref_. On the other hand, FWHM is expressed as:

10 $$ FWHM = \frac{FSR}{\pi } $$

11a $$ \text{with} \quad FSR_{LT {\text{-}} aMZI} = \frac{{\lambda^{2} }}{{2(n_{eff,sens} L_{sens} - n_{eff,ref} L_{ref} )}} $$

11b $$ \text{and} \quad FSR_{C {\text{-}} aMZI} = \frac{{\lambda^{2} }}{{(n_{eff,sens} L_{sens} - n_{eff,ref} L_{ref} )}} $$

The FWHM of LT-aMZI is half that of C-aMZI, while both increase as L_sens_ → (n_ref_/n_sens_) × L_ref_. Accordingly:

12 $$ FOM_{LT {\text{-}} aMZI} = \frac{S}{FWHM} = \frac{{2\pi S_{wg} L_{sens} }}{\lambda } = 2FOM_{C {\text{-}} aMZI} $$

FOM of LT-aMZI is double that of C-aMZI with both proportional to sensing arm length L_sens_ and its waveguide sensitivity S_wg_. In general, aMZI (or unbalanced MZI) sensor offers advantage over the sMZI. For the symmetric LT-MZI (LT-sMZI) where L_sens_ = L_ref_ = L we have:

13a $$ S_{LT {\text{-}} sMZI} = \frac{{\lambda_{res} }}{{\Delta n_{eff} }}S_{wg} $$

13b $$ FSR_{LT {\text{-}} sMZI} = \frac{{\lambda^{2} }}{{2\Delta n_{eff} L}} $$

13c $$ FOM_{LT {\text{-}} sMZI} = \frac{{2\pi S_{wg} L}}{\lambda } $$

Hence, for the LT-sMZI the sensitivity is independent of L while FWHM decrease as L increase and FOM expression is the same as in the case of LT-aMZI. However, in the asymmetric MZI sensor you can engineer both S and FOM independently using L_ref_ and L_sens_, Eq. (9) and Eq. (12), and independent of the used waveguide structures (i.e. S_wg_ and n_eff_), while in the case of symmetric MZI the sensor sensitivity is determined only by the waveguide structure, Eq. (13a). This is also an advantage over ring resonator sensors.

## Conclusion

In conclusion a LT-aMZI structure using the popular SOI technology have been proposed, this design is a compact version of the widespread C-aMZI. Four designs for optical filters and WDM applications with different FSR were implemented and characterized both numerically and experimental as well as analytically using our derived compact model. The different modelling techniques show good matching with the experimental results of the fabricated devices. It was also shown that, our LT-MZI spectrum can be tuned by changing its DCs coefficients for spectral tunable filter application. Finally, using our compact model the interferometer proved to have better performance when compared with the widespread C-MZI in sensing and modulation applications, and we also demonstrated that LT-aMZI sensor configuration is preferable over the LT-sMZI.

## Materials and methods

The designs were fabricated at the UW NNCI Washington Nanofabrication Facility using Electron Beam lithography^34^ on standard SOI wafers with silicon device layer of 220 nm. We fabricated three copies for each design to account for the fabrication tolerance.

The device measurements were performed by the team of Lukas Chrostowski at The University of British Columbia and by Maple Leaf Photonics^42,43^. A tunable laser is used to span the wavelength region from 1500 to 1600 nm. A polarization maintaining fiber array containing 4 fibers separated by 127 µm is used to couple light in and out of the chip through the grating couplers while a detector is used to measure the output power from the fibers. Also note, that the SEM photos are taken before depositing the SiO2 clad.
